# Heart Failure with Reduced Ejection Fraction—Does Sex Matter?

**DOI:** 10.1007/s11897-021-00533-y

**Published:** 2021-11-15

**Authors:** Sascha Swaraj, Rebecca Kozor, Clare Arnott, Belinda A. Di Bartolo, Gemma A. Figtree

**Affiliations:** 1 The Kolling Institute, University of Sydney, Sydney, NSW Australia; 2 Department of Cardiology, Royal North Shore Hospital, Sydney, Australia; 3 The George Institute for Global Health, Imperial College London, London, UK

**Keywords:** Heart failure, Reduced ejection fraction, Sex, Gender, Male, Female

## Abstract

**Purpose of Review:**

There is an increasing recognition of the importance of sex in susceptibility, clinical presentation, and outcomes for heart failure. This review focusses on heart failure with reduced ejection fraction (HFrEF), unravelling differences in biology, clinical and demographic features and evidence for diagnostic and therapeutic strategies. This is intended to inform clinicians and researchers regarding state-of-the-art evidence relevant to women, as well as areas of unmet need.

**Recent Findings:**

Females are well recognised to be under-represented in clinical trials, but there have been some improvements in recent years. Data from the last 5 years reaffirms that women presenting with HFrEF women are older and have more comorbidities like hypertension, diabetes and obesity compared with men and are less likely to have ischaemic heart disease. Non-ischaemic aetiologies are more likely to be the cause of HFrEF in women, and women are more often symptomatic. Whilst mortality is less than in their male counterparts, HFrEF is associated with a bigger impact on quality of life in females. The implications of this for improved prevention, treatment and outcomes are discussed.

**Summary:**

This review reveals distinct sex differences in HFrEF pathophysiology, types of presentation, morbidity and mortality. In light of this, in order for future research and clinical medicine to be able to manage HFrEF adequately, there must be more representation of women in clinical trials as well as collaboration for the development of sex-specific management guidelines. Future research might also elucidate the biochemical foundation of the sex discrepancy in HFrEF.

## Introduction

Heart failure (HF) continues to increase in prevalence in our community in men and women, estimated to affect 64.34 million people globally [1]. The prevalence of HF appears significantly higher in the female sex globally (9.16 vs. 7.69 per million inhabitants) [1]. Women are more likely than males to have HF with preserved EF (HFpEF) and are about 65% less likely to develop HFrEF [2].

There has been a growing need, and widespread call, for increased representation of women in clinical trials, for studies to be powered for sex disaggregated analyses, and for guidelines to consider and reflect sex differences. Here, we review the most recent studies and literature and summarise the sex differences in demographics, mechanism, clinical presentation, biomarkers, outcomes, clinical pathways, management and representation in the literature for HFrEF.

**Fig. 1:**
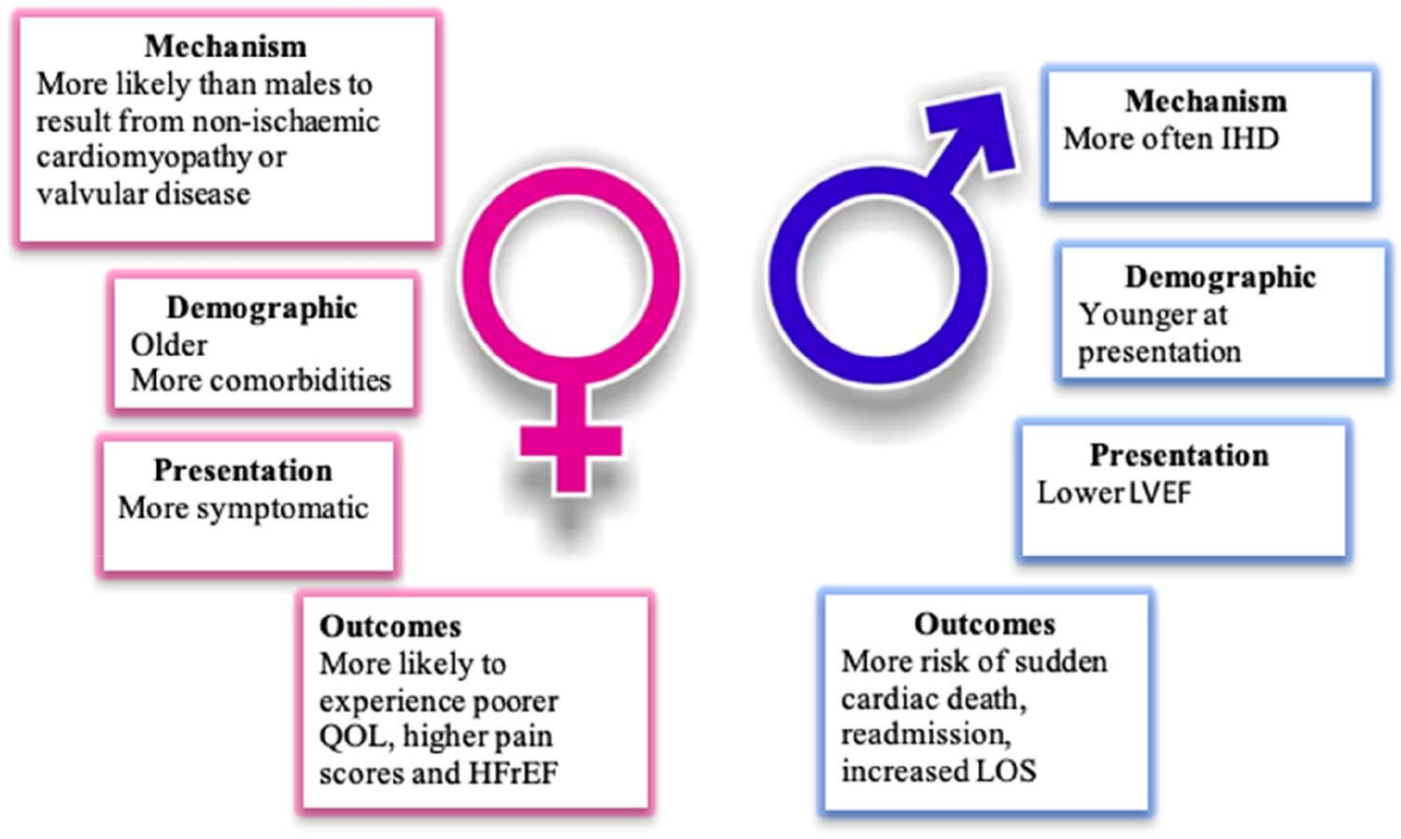
Heart failure with reduced ejection fraction—does sex matter? The impact of sex in HFrEF can be demonstrated through the mechanisms and demographics, presenting features and outcomes. Where men are more likely to develop HFrEF from ischaemic heart disease (IHD), present with lower ejection fractions and have a higher mortality, women tend to have non-ischaemic cardiomyopathy or valvular aetiology of HFrEF, be older at presentation, are more likely to have comorbidities like hypertension and chronic kidney disease, and experience more symptoms and report a poorer quality of life. QOL, quality of life; LOS, length of stay; HFrecEF, heart failure with recovered ejection fraction; IHD, ischaemic heart disease; LVEF, left ventricular ejection fraction

In addition to referring to key early studies in the field, we systematically searched databases on Embase using the search strategy (HF with reduced ejection fraction OR HFrEF) and (gender or sex or female or male), limited to the English language, humans and the years 2016 to 2021. After removing duplicates, there were 488 results. After abstracts were screened, there were 42 studies and after reading the full texts, 33 were relevant to this review, with key themes summarized in (Fig. 1).

## Clinical Demographic Differences

### Age

Whilst in both sexes the incidence of HF increases with age, it is well established that women are more likely to be older when presenting with HFrEF [3]. This is also reflected in more recent research over the last 5 years [4–6].

### Comorbidities

In general, women with HFrEF have a higher prevalence of comorbidities than men. Women with HFrEF are more likely to have hypertension, diabetes, anaemia, thyroid disease and depression, and a greater burden of chronic kidney disease (CKD) [5–7]. They are less likely to have atrial fibrillation (AF) and ischaemic heart disease as underlying contributors compared to male HFrEF patients [2, 6]. In keeping with this, they are less likely to have had myocardial infarction, percutaneous coronary intervention and coronary artery bypass grafting [4]. Diabetes is an important risk factor in HF for both sexes, but to a greater degree in women [8]. Women with HFrEF are also less likely than men to smoke [9], have a lower level of education and are more likely to be unmarried, widowed or divorced [5].

New data from the UK has found that women are more obese at presentation, and this obesity portends worse outcomes in women compared to men [5]. This may be largely due to obesity increasing the relative risk of coronary artery disease in women more than men (64% vs 46%, respectively) [2]. This however differed from a cohort of 5255 patients from an Asian registry [7] and 167 females from a Middle Eastern cohort where women with HFrEF had a lower BMI than men, with poorer outcomes, consistent with the so-called obesity paradox [10]. This data reflects the differing demographics and risk factors for women outside of western countries.

## Mechanisms

There are key sex differences in the underlying mechanisms contributing to HFrEF, as well as differences in the susceptibility to each and the relative contribution to the burden of HFrEF.

### Ischaemic

Whilst a driving precipitant of HFrEF in males is macrovascular coronary artery disease, myocardial ischaemia and infarction [11], HFrEF in women is more likely to be in association with older age, hypertension and valvular disease. Women with HFrEF are less prone than men to have macrovascular disease, including epicardial coronary artery disease or peripheral arterial disease, and stroke [3]. For example, the lifetime risk of HF in women with no history of myocardial infarction (MI) was 1 in 6, compared to 1 in 5 for all women. This likely indicates factors other than MI playing a relatively greater role in the development of HFrEF. In contrast, for men, the lifetime risk was approximately half as great in those free of MI (1 in 9) compared to all men (1 in 5), representing the heightened relevance of antecedent MI in men compared to women with HF [12].

### Non-Ischaemic

There are some HF aetiologies that are unique to women, such as peripartum cardiomyopathy, with risk factors including advanced maternal age, and pre-eclampsia [13]. Additionally, the risk of cardiomyopathy from cardiotoxicity of breast cancer adjuvant chemotherapy can be as high as 42% indicating a need for long-term follow-up in these patients [14].

In patients with underlying myocardial dysfunction and cardiomyopathy, inflammation is increasingly recognised as playing a key role in a sex-specific manner. Sex discrepancies have recently been demonstrated in antibody-mediated immune response on cardiac remodelling in HFrEF, affecting progression of HF. We know that IgG can be found in the myocardium in patients with end-stage HFrEF associated with ischaemic heart disease (IHD). One study indicated that, in the early stages of remodelling, IgG1 and IgG3 levels differ between men and women [15]. Further research into implications of this is required to elucidate if inflammation in IHD confers a worse prognosis for remodelling, especially after an MI, whether this differs between sexes, and the implication for treatment.

Takotsubo cardiomyopathy is also more common in postmenopausal women. Whilst classically understood to be an acute and reversible condition, there is increasing appreciation of longer-term myocardial abnormalities and many patients continue to suffer symptoms which may be attributed to the recovery process [16, 17].

More evidence is emerging on the impact of sex hormones on sex discrepancy in HFrEF. There is increasing research on the protective effects of oestrogen on HF. Additionally, an oestrogen depletes postmenopausal state may contribute to women having higher left ventricular systolic and diastolic stiffness when compared to men as oestrogen is involved in blood pressure and arterial tone regulation [18]. Furthermore, the recent genetic variant analysis revealed the positive association between endogenous testosterone and HF [19, 20].

## Clinical Presentations

In terms of presenting features of HFrEF, most recent data concurred with older data [7, 21] showing that women are more likely to experience symptoms like dyspnoea, exhibit a third heart sound (S3 gallop), increased jugular venous pressure, and leg oedema when compared to men. One paper conflicted with this but had a smaller sample size of 118 women and was restricted to a CCU setting [4]. It was reported that women experienced less symptoms with more normal ECGs.

Echocardiography analysis highlights that women have higher ejection fractions with smaller left atria, higher longitudinal strain and higher circumferential strain [4, 22]. Asian women in particular more commonly have concentric left ventricular geometry compared to male counterparts [7]. Conversely, a large study looking at 12,058 men and 3357 women showed no difference in left ventricular ejection fraction between sexes [6]. Furthermore, whilst left ventricular (LV) systolic function is an important predictor of mortality in men, right ventricular function and LV diastolic function better predicted mortality in women [22]

As coronary microvascular and macrovascular dysfunction have similar risk factors, unique biological risk factor profiling is a promising method of early diagnosis [23]. Currently, this is quite limited, however, by discovering new mechanisms and exploring underlying aetiologies at a molecular level, more avenues may arise for future trial design to realise gender-specific guidelines. This could give rise to phenotyping of HF with sex differentiation. Recent data has been able to identify phenotypes of HFrEF via clustering variables like history of coronary artery bypass or percutaneous coronary intervention (PCI), sex, CKD, race, etc., and show different mortality associated with each [24]. Further phenotyping with molecular level variables derived from omics analysis could elucidate sex-based differentiation of phenotypes as well.

## Biomarkers

Biomarker identification in HF could identify potential targets for HF prevention in higher-risk individuals [25]. One biomarker already utilised is B-type natriuretic peptide (BNP), which has proven to be predictive of in-hospital mortality for both women and men in all types of HF [3]. Three studies explored serum biomarkers and their differences between the sexes. One showed similar levels of N-terminal pro-BNP [6] between men and women, whilst another study showed women tended to have a higher BNP [26]. Although the implications are unknown currently, one paper discovered that high plasma concentrations of beta hydroxybuturate were associated with increased risk of HFrEF particularly in women [27].

Importantly, data from 12 years’ worth of follow-up, after adjustment of risk factors, showed that a more androgenic hormone profile among post-menopausal women was associated with increased risk of HF [28]. Whilst exogenous hormone therapy in post-menopausal women is not beneficial for primary prevention, measuring endogenous hormone levels could be useful for risk stratification especially in populations known to have more androgenic profiles like in polycystic ovarian syndrome. In addition, there is evidence to also support the use of hsTroponin I alongside BNP in predicting new-onset HFpEF with similar reported effects in men and women [29, 30]. Once risk of HFpEF is established, the same study also found that galectin-3 measurements could help monitor its progression over time. These, whilst promising, seem unlikely to be affected by sex or the presence of sex hormones highlighting the need for further evidence into varying biomarkers between HFrEF and HFpEF to aid in clinical diagnosis and management related to sex.

## Clinical Pathways

International guidelines such as those of the European Society of Cardiology [31] or the American College of Cardiology [32] have provided clear pathways for initial investigations and subsequent treatment in patients presenting with HFrEF based on large-scale clinical trials. However, many of the clinical trials have a challenge with under-representation of females [33].

## Management

### Medical Therapy

Numerous recent studies have demonstrated that women are less likely to receive evidence-based medical therapy for treatment of HFrEF compared to their male colleagues [5, 6, 34]. Recent data from two large HFrEF trials (PARADIGM-HF and AT-MOSPHERE) has identified under prescribing of diuretics, anticoagulants and device therapy in women with HFrEF compared to men [6]. A major advance in the pharmacotherapy treatment for HFrEF has been the introduction of the angiotensin receptor-neurolysin inhibitor combination (ARNI). However, women have been observed to be less likely to receive appropriate prescription, with some studies showing female gender to be independently associated with lower odds of ARNI prescription [34, 35]. This data is even more striking in the context subgroup analysis from the PROVE-HF study showing that, when compared to men, women had a more rapid and early reduction in serum N-terminal pro-BNP after ARNI initiation [36]. Furthermore, whilst women and men had similar degrees of reverse left ventricular remodelling after ARNI use, women displayed earlier and more consistent remodelling. The reports of lower rates of ARNI prescription, and lower diuretics, anticoagulants and device therapy contrast with reports of equal adherence to renin-angiotensin system inhibitors (ACEi), beta blockers, mineralcorticoid receptor antagonists and ivabradine, at least in the predominantly male study population of one study [5]. Additionally, a recent meta-analysis consisting of 13,833 patients, of which 24% were women, confirmed that, in patients with HFrEF in sinus rhythm, β-blockers reduced all-cause mortality and HF admissions, irrespective of age or sex [37].

Differences in tolerability and efficacy between drug therapies provide evidence that women require different guidelines for medical therapy of HFrEF [11, 38]. For example, in women with HFrEF, the maximum drug benefit of a beta blocker is reached at 50–60% of the guideline-recommended dosing, decreasing the risk of all-cause death and HFrEF associated hospitalisation by 30% [11]. Similarly, for an ACEi, 40–60% of a standard dose is enough to reach efficacy in women, resulting in a 30% risk reduction. If women experience twice the rate of adverse events from HF drug regimes, as the literature suggests [11], there is a clear impetus for sex-based dosing targets and guidelines. Additionally, with digoxin use, plasma concentrations in women were higher than men at guideline dosing, increasing the risk of digoxin-related mortality. Women in their patient population were older with lower body weight and height [35], and these sex differences can effect pharmacokinetics and pharmacodynamics. Guidelines that inform drug dosing are often reliant on research with limited female participation [39]. Further clinical research is needed to confirm the presence of differential dose efficacy between sexes and then define sex-specific, and even age-specific optimal dosing.

### Device Therapy

Advances in electric and mechanical devices have continued with substantial benefits for patient symptoms, hospitalisation and outcomes, with strong evidence in both sexes [40] (Table 1). The range of devices includes implantable cardioverter defibrillators (ICD), cardiac resynchronisation therapy (CRT) and cardiac resynchronisation therapy defibrillators. However, recent data shows women are less likely to receive an ICD, and when they do, they have higher rates of implantation-related complications like pneumothorax and infection [11].

**Table 1:** Guideline-based therapy application in females with HFrEF

	Specific differences in benefits between sexes	Likelihood of getting evidence-based treatment	Dose adjustment required
Diagnostic pathway			
BNP	No [3]	-	-
TTE	No [22]	-	-
Pharmacotherapy			
ACEI/ARB	No [11]	-	Yes [38]
Betablocker	No [5]	Under prescribed [5]	Yes [5]
Digoxin	No [11]	-	Yes [11]
Diuretics	-	Under prescribed [6]	-
Anticoagulants	^−^	Under prescribed [6]	-
ARNI	No [36]	Under prescribed [35]	-
Device therapy			
ICD	No [5]	Underused [6]	-
CRT	Women more likely to respond [11]	More than male equivalent [11]	-
Cardiac rehab	No [11]	Under enrolled [11]	-

*BNP* B-type natriuretic peptide, *TTE* transthoracic echocardiogram, *ACEI* angiotensin-converting enzyme inhibitors, *ARB* angiotensin II receptor blockers, *ARNI* angiotensin receptor neprilysin inhibitor, *ICD* implantable cardioverter-defibrillator, *CRT* cardiac resynchronization therapy, *HFrEF* heart failure reduced ejection fraction.

### Cardiac Rehabilitation and Lifestyle Modifications

There is strong evidence, reflected in the guidelines, for cardiac rehabilitation (including education), and lifestyle modifications including salt reduction, weight loss, and exercise (strong evidence) and a whole grain plant-based diet (less evidence) [41], in improving quality of life and outcomes in patients with HFrEF [42]. Disappointingly, women are seen to have lower enrolment and completion of cardiac rehabilitation, and this may be due to sex-specific discrepancies in presenting factors like being older, with more comorbidities and less social supports [11]. Ultimately, the female gender is associated with being less likely to receive optimal treatment as recommended by guidelines [43].

## Outcomes

The literature reports on multiple outcomes in HF, exploring mortality, quality of life, rates of hospitalisation and readmission, susceptibility to arrhythmia, and recovery of LV function. The previous body of evidence found no difference in-hospital mortality between sexes, which also correlated with several recent studies [4, 44]. A few recent studies reported a lower rate of mortality in women [6, 45], with one also reporting that sudden cardiac death occurred more often in males with HFrEF [46]. A recent prospective multicentre cohort study showed that women with ischaemic heart disease and HFrEF had a lower survival rate than women without IHD (*p* = 0.001), and this survival difference was not observed in men [47].

Previous literature had described that females with HF report lower quality of life, and this was reiterated in new data [6]. Despite females living longer than their male counterparts with HFrEF, they experienced more symptoms and signs, ultimately had a poorer quality of life, higher pain scores and greater self-reported psychological and physical disability [48]. Similarly, when Kansas City Cardiomyopathy Questionnaire (KCCQ) scores of patients with HFrEF were compared, female patients scored 1.8 points lower than males [49]. In terms of another morbidity, new data from a study that surveyed patients after PCI showed that, at 30 days follow-up, rates of arrhythmia and recurrent MI were higher in women with HFrEF compared to men [24].

Regarding rates of hospitalisations, most data, including more recent studies, showed that women have a lower risk of hospitalisation [6]. Only one recent paper showed women had more hospitalisations than men, albeit reporting on a mostly male population (70.1%) [5]. Additionally, papers reviewed in the last 5 years also indicated women were less likely to be readmitted for HF and had a shorter length of stay [4, 50, 51].

Although many studies have explored predictive factors for HF with recovered ejection fraction, few have sought to understand whether there is a gender discrepancy. More recent data has identified a link between female sex and left ventricular ejection fraction recovery [52–54]. One retrospective cohort study elucidated specific clinical phenotypes and predictors for ejection fraction recovery and showed that females were more likely to recover (10.1% vs 14%, *p* = 0.04) [48]. Since patients with recovered ejection fraction have a much better prognosis compared to those with HFrEF, female patients in these studies also exhibited a lower mortality risk compared to men. Regarding complications, whilst diabetes confers a worse quality of life in patients with HFrEF irrespective of sex, there is a greater risk of adverse outcomes in women than men with diabetes [8].

An important consideration in the interpretation of the literature in regard to heart failure outcomes is the often-poor stratification of analyses and reports by acute versus ambulant or chronic heart failure, with less data available regarding sex differences in the acute heart failure setting. Data that is available suggests that, whilst co-morbidities at the time of presentation differ between the sexes, acute in-hospital outcomes are similar. For example, in the ALARM-HF multinational survey of over 4000 patient hospitalizations, men were more likely to have acute coronary syndrome as the precipitating event and underlying coronary artery disease, and women were more likely to have concomitant AF, obesity, anaemia and depression. In hospital, mortality, however, was similar in both sexes (11%) [55]. This contrasts with the lower mortality risk more broadly observed in women with heart failure discussed above.

## Sex Representation in Literature

Four studies closely analysed and quantified female representation in trials. Female participation in landmark HF trials ranged from 0 to 40% with an average of 20% [26]. In an analysis of 118 HF trials, 58,873 of 215,508 people enrolled were women (27% on average), improving from 26% in 2001–2004 to 29% in 2013–2016 [33]. Another study found that females represented only 25.5% of enrolled participants with HFrEF (ranging from 4 to 68% in each trial) when looking at 317 randomised control trials. Another explanation for poor female representation relative to how many females are affected by HF may be due to 81 of those studies using sex-specific exclusion criteria (e.g., childbearing, menopausal status) without explanation. As a result, among these 81 studies, only 23.3% of the sample population was female [56].

Under representation of females in clinical trials and lack of sex-disaggregated data means studies are often underpowered to apply results to clinical practice. For more than 25 years, there has been an acknowledgement of the sex discrepancy in HFrEF [57], and despite this, the underrepresentation of women in clinical trial data continues. The lack of sex disaggregation perpetuates the inability for sex-specific treatment and diagnostic guidelines.

## Conclusion

The sex differences in HF with reduced ejection fraction are undeniable. The pathophysiology, types of presentation, morbidity and mortality differ between the sexes. However, without more representation of women in clinical trials, the development of sex-specific management guidelines appears improbable.
